# Multiomics and Genomic Alteration Characterization Identifies VDAC1 as a Mitochondrial‐Associated Biomarker in Pancreatic Cancer

**DOI:** 10.1155/humu/5510306

**Published:** 2026-05-06

**Authors:** Wanliang Sun, Yifei Wang, Xuxu Qiao, Shuo Zhou, Zhong Liu, Song Yang, Dengyong Zhang, Dongdong Wang, Hu Zhou, Feiyu Qi, Mingrui Zhang, Binquan Wu, Yi Tan, Lijun Cen, Biao Zhang

**Affiliations:** ^1^ Department of Hepatopancreatobiliary Surgery, The First Affiliated Hospital of Bengbu Medical University, Bengbu, China, bbmc.edu.cn; ^2^ Department of Cardiology, The First Affiliated Hospital of Bengbu Medical University, Bengbu, China, bbmc.edu.cn; ^3^ Department of Medical Oncology, The First Affiliated Hospital of Bengbu Medical University, Bengbu, China, bbmc.edu.cn; ^4^ School of Medicine, Nankai University, Tianjin, China, nankai.edu.cn; ^5^ School of Medicine, Bengbu Medical University, Bengbu, China, bbmc.edu.cn; ^6^ Department of Transfusion Medicine, Affiliated Hospital of Youjiang Medical University for Nationalities, Baise, China, gxyyfy.cn; ^7^ Key Laboratory of Molecular Pathology in Tumors of Guangxi Higher Education Institutions, Affiliated Hospital of Youjiang Medical University for Nationalities, Baise, China, gxyyfy.cn

**Keywords:** copy number variation, genomic alterations, mitochondrial metabolism, multiomics integration, pancreatic ductal adenocarcinoma, prognostic biomarker, VDAC1

## Abstract

Pancreatic ductal adenocarcinoma (PDAC) is characterized by aggressive biological behavior and limited therapeutic responsiveness. Identifying genetically and biologically interpretable biomarkers remains critical for improving molecular stratification of this malignancy. Here, we performed an integrative analysis combining bulk transcriptomics, genomic alteration profiling, immune‐response modeling, and functional validation to characterize the clinical relevance of voltage‐dependent anion channel 1 (VDAC1) in PDAC. Genomic analyses demonstrated that VDAC1 exhibits a low somatic mutation frequency in PDAC and that its dysregulated expression may be partly associated with copy number alterations. Transcriptomic analyses further showed that VDAC1 is significantly upregulated in tumor tissues and associated with unfavorable clinical outcomes. Multiomics characterization indicated that high VDAC1 expression is linked to mitochondrial metabolic programs and reduced cytotoxic immune signatures. Functional validation demonstrated that VDAC1 silencing impaired pancreatic cancer cell proliferation and disrupted mitochondrial homeostasis, including reduced mitochondrial membrane potential, ATP production, and mitochondrial reactive oxygen species levels. In addition, VDAC1 perturbation reduced immunosuppressive cytokine output, suggesting a tumor‐intrinsic connection between mitochondrial metabolic regulation and immune‐related programs. Collectively, this study provides a genomic alteration‐informed multiomics framework supporting VDAC1 as a mitochondrial‐associated biomarker in PDAC and highlights its potential relevance for molecular characterization of pancreatic cancer.

## 1. Introduction

Pancreatic cancer has become an increasingly urgent global health problem, with a fatality burden that remains disproportionately high relative to incidence [1]. In 2022, an estimated 510,992 new cases and 467,409 deaths were recorded worldwide, illustrating the persistently narrow gap between diagnosis and mortality driven by late detection and limited durability of available therapies [2]. In the United States, pancreatic cancer is projected to remain among the leading causes of cancer‐related death, reinforcing the need to define tractable biological dependencies that can be leveraged to achieve durable disease control rather than transient responses [3].

A central feature that underlies therapy refractoriness in pancreatic ductal adenocarcinoma (PDAC) is the unique composition and organization of its tumor microenvironment (TME) [4–6]. Malignant epithelial programs are embedded within a desmoplastic ecosystem enriched for activated fibroblast/stellate lineages, aberrant vasculature, and immune populations that are frequently skewed toward suppressive myeloid and dysfunctional lymphoid states [7, 8]. Importantly, this architecture is not a passive scaffold: bidirectional signaling between tumor cells and stromal compartments governs tissue mechanics, restricts drug delivery, and shapes immune recruitment, priming, and effector function [9]. As a consequence, immune failure in PDAC is increasingly interpreted as an emergent, systems‐level property of microenvironmental wiring—where tumor‐intrinsic signals, stromal barriers, and immune‐context cues converge—rather than an isolated defect within any single compartment.

Metabolic reprogramming is a powerful mechanism by which tumors can actively engineer such microenvironmental states [10]. Beyond fueling proliferation, mitochondrial bioenergetic and redox programs can alter nutrient competition, stress‐adaptation signaling, and cytokine/chemokine landscapes, thereby resculpting immune composition and suppressing cytotoxic antitumor activity [11, 12]. This metabolic–immune coupling is particularly relevant in PDAC, where nutrient limitation, hypoperfusion, dense stroma, and therapy‐induced stress intensify selective pressures for mitochondrial‐centered adaptations that simultaneously enhance tumor fitness and reinforce immune restriction [13]. From the standpoint of treatment resistance, these adaptations matter not only because they support survival under cytotoxic stress, but also because they can stabilize an immune‐excluded or immune‐dysfunctional TME that undermines durable control.

Voltage‐dependent anion channel 1 (VDAC1) occupies a strategic control point within this axis [14, 15]. As a principal pore of the mitochondrial outer membrane, VDAC1 regulates the exchange of key metabolites and contributes to stress and redox signaling, positioning it to influence survival programs, cell‐state transitions, and adaptive phenotypes under hostile microenvironmental conditions [15, 16]. Despite this plausibility, it remains insufficiently defined in PDAC how VDAC1 relates to TME organization and whether VDAC1‐associated mitochondrial remodeling manifests as coordinated immune suppression and therapy‐refractory phenotypes in clinically relevant contexts.

Here, we integrate bulk transcriptomic profiling, genomic alteration characterization, and immune‐response modeling to delineate the relationship between VDAC1 and PDAC biology. We then anchor these computational observations through functional validation in pancreatic cancer cell lines, focusing on mitochondrial homeostasis (mitochondrial membrane potential, ATP production, and mitochondrial reactive oxygen species), together with immune‐facing tumor programs and soluble immunoregulatory outputs (e.g., checkpoint‐ and antigen‐presentation–related markers and immunosuppressive cytokines). By placing VDAC1 at the intersection of mitochondrial metabolic remodeling and TME immune restriction, we aim to establish a mechanistically interpretable biomarker framework for understanding PDAC progression.

## 2. Methods

### 2.1. Target Collection and Disulfiram‐Guided Intersection Analysis

Disulfiram was used as a drug‐informed chemical probe to nominate pharmacologically tractable candidate nodes rather than to claim therapeutic efficacy. The two‐dimensional structure of disulfiram was retrieved from PubChem [17]. Putative disulfiram targets were compiled from multiple public resources, including CTD, STITCH, SwissTargetPrediction, and SEA [18–22]. Pancreatic adenocarcinoma (PAAD)–associated genes were obtained from GeneCards and OMIM; GeneCards entries were filtered using a relevance score ≥ 15 to prioritize disease‐relevant candidates [23, 24]. Intersecting genes between the disulfiram target set and the PAAD‐associated gene set were identified and visualized using Sangerbox [25].

### 2.2. Protein–Protein Interaction (PPI) Network and Functional Enrichment

PPI information for intersecting genes was retrieved from STRING, retaining only high‐confidence interactions (combined score ≥ 0.995) to reduce spurious edges [26]. The resulting PPI network was imported into Cytoscape (v3.9.0) for visualization and network‐based analyses [27]. Gene Ontology (GO) and KEGG enrichment analyses were performed for the intersecting targets, with significance defined as *p* < 0.05 unless otherwise stated [28, 29].

### 2.3. VDAC1‐Centered Subnetwork Extraction and Hypothesis‐Generating Docking

To focus on a mechanistically interpretable hub, a VDAC1‐centered local subnetwork was extracted by retaining VDAC1 and its first‐order interacting partners from the global PPI network. Molecular docking between disulfiram and VDAC1 was conducted as a hypothesis‐generating analysis using CBDOCK2 [30]. The VDAC1 protein structure was obtained from the Protein Data Bank, and the disulfiram ligand structure was obtained from PubChem. Docking poses were visualized using PyMOL [31]. Docking analyses were used to explore structural compatibility and do not constitute experimental validation of binding.

### 2.4. Bulk Cohorts and Data Acquisition

Bulk RNA‐seq and clinical annotations for PAAD were downloaded from the UCSC Xena data hubs using uniformly processed TCGA/GTEx resources [32]. The bulk dataset included 179 TCGA‐PAAD tumor samples and 4 TCGA adjacent/solid‐tissue normal samples, together with 167 normal pancreas samples from GTEx for tumor–normal comparison [33, 34]. Expression matrices were integrated with clinicopathological annotations at the sample level. Clinical outcome endpoints—overall survival (OS), disease‐specific survival (DSS), disease‐free interval (DFI), and progression‐free interval (PFI)—were derived from standardized TCGA outcome annotations when available [35]. For each endpoint, samples lacking the corresponding time‐to‐event or event indicator were excluded in an endpoint‐specific manner.

### 2.5. Prognostic Modeling and Survival Analyses

Prognostic modeling was performed using LASSO Cox regression implemented in the glmnet R package with 10‐fold cross‐validation [36]. A risk score for each patient was calculated using the linear predictor formed by the expression levels and coefficients of features with nonzero weights [37]. Survival differences for OS, DSS, DFI, and PFI were evaluated using Kaplan–Meier curves and log‐rank tests [38]. Where results across endpoints or cohorts required aggregation, meta‐analysis was performed using a random‐effects model [39].

### 2.6. Bulk Analyses of VDAC1 Expression and Clinical Associations

VDAC1 expression in PAAD tumor tissues and nontumor pancreatic tissues was assessed using TCGA‐PAAD and GTEx normal pancreas RNA‐seq data accessed via UCSC Xena. Diagnostic discrimination between tumor and nontumor samples was evaluated by receiver operating characteristic (ROC) curve analysis, and the area under the curve (AUC) was calculated [40]. For prognostic evaluation, patients were stratified into high‐ and low‐VDAC1 groups using the median expression value unless otherwise specified; Kaplan–Meier survival analyses were performed for OS, DSS, DFI, and PFI with significance assessed by log‐rank testing. Associations between VDAC1 expression and clinicopathological variables were tested using nonparametric methods appropriate to variable type (e.g., Wilcoxon rank‐sum test for two‐group comparisons and Kruskal–Wallis test for multigroup comparisons).

VDAC1 expression across immune subtypes was evaluated by matching TCGA‐PAAD samples to the published TCGA pan‐cancer immune subtype classification (C1–C6) [41]. Cell‐cycle phase annotations were derived using canonical G1/S and G2/M gene signatures with a standard gene set–based scoring approach; VDAC1 expression differences across phases were assessed using the Kruskal–Wallis rank‐sum test [42].

### 2.7. Pathway Enrichment and Variation Analyses Associated With VDAC1 Expression

To characterize pathway‐level programs associated with VDAC1, TCGA‐PAAD samples were stratified by VDAC1 expression using percentile‐based extremes (top 30% as high and bottom 30% as low) to increase contrast for enrichment analyses. Differential expression statistics between groups were used to generate ranked gene lists for gene set enrichment analysis (GSEA) [43]. Hallmark gene sets were used as the primary collection [44], and enrichment robustness was evaluated using additional curated resources including GO, Reactome, and WikiPathways; normalized enrichment scores (NES) and adjusted *p* values were used for significance assessment where applicable [45, 46].

Gene set variation analysis (GSVA) was performed using the GSVA R package with the *z*‐score algorithm to quantify sample‐wise pathway activity [47]. Metabolic pathway gene sets were obtained from KEGG, and between‐group differences in GSVA scores were assessed using the limma package [48]. To assess cross‐dataset consistency, oncogenic signature GSVA was additionally evaluated in an independent GEO cohort.

### 2.8. Immune‐Related Analyses Associated With VDAC1 Expression

Immune features associated with VDAC1 expression were quantified using TCGA‐PAAD bulk RNA‐seq data. Immune response scores—including methylation‐based tumor‐infiltrating lymphocyte score (MeTIL), cytolytic activity (CYT), interferon‐*γ*–related signature, tertiary lymphoid structure (TLS) signature, T cell–inflamed signature, and chemokine‐associated signatures—were computed using the easier R package [49]. Samples were stratified into high and low VDAC1 expression groups by median expression, and differences were assessed using the Wilcoxon rank‐sum test.

To infer upstream immune signaling activities, pathway‐level scores were computed using the PROGENy framework implemented within easier; pathway scores were standardized as *Z*‐scores prior to downstream analyses [50]. Cancer–immunity cycle activities were quantified using predefined scores from the TIP framework, and associations with VDAC1 expression were evaluated using Spearman correlation analysis [51]. Immune cell infiltration estimates were obtained using TIMER2.0 [52]. Correlations between inferred immune cell abundance and VDAC1 expression were assessed using Spearman correlation, and group differences between VDAC1‐high and VDAC1‐low samples were evaluated using the Wilcoxon rank‐sum test.

### 2.9. Drug Sensitivity Correlation Analysis

Drug sensitivity profiles were obtained from CTRP v2.0 and the PRISM Repurposing datasets, where response is represented by the area under the dose–response curve (AUC), with lower AUC indicating higher sensitivity [53, 54]. Compounds with > 20% missing AUC values were excluded, and the remaining missing values were imputed using k‐nearest neighbor (k‐NN) imputation [55]. Because cell lines in both datasets are derived from the Cancer Cell Line Encyclopedia (CCLE), matched CCLE gene expression profiles were used for correlation analyses [56]. Associations between VDAC1 expression and drug AUC values were evaluated using Spearman correlation. Positive correlations were interpreted as higher VDAC1 expression being associated with relative resistance (higher AUC), whereas negative correlations indicated relative sensitivity. Drugs were ranked by statistical significance (*p* value), and the Top 30 compounds were visualized using lollipop plots.

### 2.10. Genomic Alteration and Copy Number Variation Analysis of VDAC1 in PAAD

The genomic alteration and copy number variation characteristics of VDAC1 in PAAD were analyzed using publicly available TCGA‐PAAD datasets. The overall copy number alteration landscape of the PAAD cohort was assessed based on GISTIC scores. The alteration frequency of VDAC1 was summarized to evaluate its mutation prevalence in PAAD. To further investigate whether copy number variation might contribute to VDAC1 dysregulation, the association between VDAC1 copy number status and mRNA expression was examined. VDAC1 expression distributions across different copy number states were compared, and the correlation between GISTIC2 copy number values and normalized mRNA expression levels was evaluated using Spearman correlation analysis. In addition, differences in VDAC1 expression among copy number subgroups were assessed using the Kruskal–Wallis test. A two‐sided *p* value < 0.05 was considered statistically significant.

### 2.11. Cell Culture

Human PDAC cell lines PANC‐1 (RRID: CVCL_0480) and MIA PaCa‐2 (RRID: CVCL_0428) were used. PANC‐1 and MIA PaCa‐2 cells were obtained from ATCC. Cells were cultured in Dulbecco′s Modified Eagle′s Medium (DMEM) supplemented with 10% fetal bovine serum (FBS) and 1% penicillin‐streptomycin at 37°C in a humidified incubator with 5% CO_2_. Cells were routinely tested for mycoplasma contamination and passaged for no more than 15 generations prior to experimentation.

### 2.12. Construction of Stable shVDAC1 Knockdown Cell Lines

Stable VDAC1 knockdown pancreatic cancer cell lines were generated using lentiviral short hairpin RNA (shRNA) constructs. Three independent shRNAs targeting VDAC1 (shVDAC1‐1, shVDAC1‐2, and shVDAC1‐3) and a nontargeting control (shNC) were obtained from GeneChem (Shanghai, China). PANC‐1 and MIA PaCa‐2 cells were infected with lentiviral particles in the presence of polybrene according to the manufacturer′s protocol. After infection, cells were selected with puromycin (2 *μ*g/mL) for 5–7 days to establish stable knockdown lines. Knockdown efficiency was validated by quantitative real‐time PCR. All three shVDAC1 constructs showed effective suppression of VDAC1 expression and were used in subsequent functional assays to ensure robustness of the observed phenotypes.

### 2.13. Quantitative Real‐Time PCR

Total RNA was extracted using TRIzol reagent (Thermo Fisher Scientific, Cat. No. 15596018) following the manufacturer′s protocol. RNA concentration and purity were assessed spectrophotometrically. cDNA was synthesized using PrimeScript RT Reagent Kit (Takara, Cat. No. RR037A). Quantitative PCR was performed using TB Green Premix Ex Taq II (Takara, Cat. No. RR820A) with GAPDH as an internal control. Relative mRNA expression was calculated using the 2^−*ΔΔ*Ct^ method. Experiments were performed in triplicate and repeated independently at least three times. The primer sequences are as shown in Table S1.

### 2.14. Cell Colony Formation

Colony formation assays were used to assess long‐term proliferative capacity. Stable shNC and shVDAC1 cells were seeded into six‐well plates at low density and cultured for 10–14 days until visible colonies formed. Colonies were washed with PBS, fixed with 4% paraformaldehyde, stained with crystal violet, and counted manually (colonies > 50 cells). Colony counts were normalized to the shNC control. Experiments were independently repeated at least three times.

### 2.15. Cell Viability Assay (CCK‐8)

Cell viability was quantified using the Cell Counting Kit‐8 (CCK‐8). Stable shNC and shVDAC1 (shVDAC1‐1/2/3) PANC‐1 and MIA PaCa‐2 cells were seeded into 96‐well plates and measured at 0, 24, 48, 72, and 96 h. At each time point, CCK‐8 reagent was added according to the manufacturer′s protocol, and absorbance at 450 nm was measured using a microplate reader. Viability was expressed as relative absorbance normalized to shNC controls. Each experiment included technical replicates and was repeated independently at least three times.

### 2.16. Measurement of Mitochondrial Membrane Potential (TMRM)

Mitochondrial membrane potential (*ΔΨ*m) was assessed by tetramethylrhodamine methyl ester (TMRM; Thermo Fisher Scientific, T668) staining and flow cytometry. Stable shNC and shVDAC1 PANC‐1 and MIA PaCa‐2 cells were incubated with TMRM at 37°C in the dark per the manufacturer′s instructions, washed with PBS, and analyzed immediately by flow cytometry. TMRM fluorescence was detected in the PE channel (excitation/emission ≈488/585 nm). Forward scatter (FSC) was used to gate viable cells. *ΔΨ*m was quantified by fluorescence intensity and normalized to shNC controls. Experiments were repeated independently at least three times.

### 2.17. Measurement of Intracellular ATP Levels

Intracellular ATP was measured using the Cell‐ATP Viability Detection Kit (MCE, Cat. No. HY‐K0302). Stable shNC and shVDAC1 PANC‐1 and MIA PaCa‐2 cells were seeded into 96‐well plates. At the indicated time points, cells were lysed and ATP detection reagent was added; luminescence was recorded using a microplate reader. ATP levels were normalized to shNC controls and reported as relative ATP. Experiments included technical replicates and were repeated independently at least three times.

### 2.18. Measurement of Mitochondrial ROS (mtROS) (MitoSOX Red)

mtROS was assessed using MitoSOX Red (Thermo Fisher Scientific, Cat. No. M36005) and flow cytometry. Stable shNC and shVDAC1 PANC‐1 and MIA PaCa‐2 cells were stained with MitoSOX Red at 37°C in the dark, washed, and analyzed immediately. Fluorescence was detected in the PE channel (excitation/emission ≈488/580 nm), and FSC gating was used to restrict analysis to viable cells. mtROS levels were quantified by fluorescence intensity and normalized to shNC controls. Experiments were repeated independently at least three times.

### 2.19. Enzyme‐Linked Immunosorbent Assay (ELISA)

Secreted immunosuppressive cytokines transforming growth factor‐*β* (TGF‐*β*) and interleukin‐10 (IL‐10) were quantified in culture supernatants by ELISA. Stable shNC and shVDAC1 cells were cultured under standard conditions; supernatants were collected and centrifuged to remove debris. Human TGF‐*β*1 and IL‐10 levels were measured using commercial kits (R&D Systems: Human TGF‐*β*1 DuoSet ELISA, Cat. No. DY240; Human IL‐10 DuoSet ELISA, Cat. No. DY217B) according to manufacturers′ instructions. Absorbance was measured using a microplate reader, cytokine concentrations were calculated from standard curves, and values were normalized to shNC controls. Experiments included technical replicates and were repeated independently at least three times.

### 2.20. Statistical Analysis

Statistical analyses were performed using R (version ≥ 4.1.0) and GraphPad Prism as appropriate. Two‐group comparisons were conducted using the Wilcoxon rank‐sum test or Student′s *t* test as specified in figure legends and method subsections. For comparisons across more than two groups, the Kruskal–Wallis rank‐sum test was used. Survival differences were evaluated using Kaplan–Meier analysis with log‐rank testing. Correlation analyses were performed using Spearman′s rank correlation coefficient. Multiple testing correction was applied for pathway‐ and gene set–based analyses where appropriate, and adjusted *p* values were used to determine statistical significance. Unless otherwise specified, in vitro experiments were independently repeated at least three times; data are presented as mean ± standard deviation (SD) or median with interquartile range as indicated. A two‐sided *p* value < 0.05 was considered statistically significant.

## 3. Results

### 3.1. Disulfiram‐Guided Nomination of Candidate Nodes and Functional Landscape in PAAD

**Figure 1:**
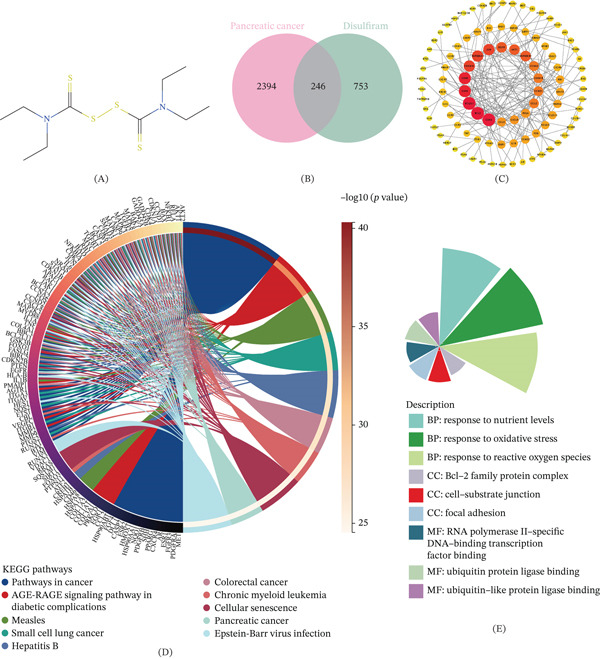
Disulfiram‐guided nomination of candidate targets and functional landscape in pancreatic adenocarcinoma. (A) Two‐dimensional chemical structure of disulfiram. (B) Venn diagram showing the overlap between pancreatic cancer‐related genes and disulfiram‐associated targets, identifying 246 shared genes. (C) Protein–protein interaction (PPI) network of the intersecting genes. (D) KEGG pathway enrichment analysis of the intersecting genes shown as a chord diagram. (E) Gene Ontology (GO) enrichment analysis of the intersecting genes across biological process (BP), cellular component (CC), and molecular function (MF) categories.

The chemical structure of disulfiram is shown in Figure 1A. Disulfiram was used here as a drug‐informed chemical probe to nominate pharmacologically tractable candidate nodes rather than to assert therapeutic efficacy in PAAD. By integrating multisource drug‐target resources, we curated 753 putative disulfiram‐associated targets. In parallel, 2394 PAAD‐related genes were retrieved after disease‐relevance filtering. Intersection analysis identified 246 shared genes (Figure 1B), which were retained as a candidate set for downstream analyses. A high‐confidence PPI network constructed from these intersecting genes showed dense interconnectivity (Figure 1C), suggesting that these candidates may participate in coordinated biological processes. KEGG enrichment analysis indicated that these genes were mainly enriched in cancer‐related pathways, including pathways in cancer, pancreatic cancer, cellular senescence, and several other disease‐associated signaling pathways (Figure 1D). GO enrichment analysis further showed significant enrichment in biological processes related to response to nutrient levels, response to oxidative stress, and response to reactive oxygen species; cellular components including the Bcl‐2 family protein complex, cell‐substrate junction, and focal adhesion; and molecular functions such as RNA Polymerase II‐specific DNA‐binding transcription factor binding, ubiquitin protein ligase binding, and ubiquitin‐like protein ligase binding (Figure 1E). Together, these findings define the functional landscape of the intersecting candidate genes and provide a basis for subsequent prioritization of key nodes for further analysis.

### 3.2. Prioritization of VDAC1 as a Mitochondrial Control Node Within the Nominated Network

Guided by network topology and enrichment patterns, we prioritized VDAC1 for further analysis as a candidate node potentially linking mitochondrial regulation with downstream cellular programs. A VDAC1‐centered local interaction network showed first‐order connections with proteins related to apoptosis regulation and mitochondrial homeostasis, including BCL2L1, BCL2, HSPA9, and SOD1 (Figure S1A), supporting a potential role of VDAC1 at the interface between mitochondrial function and cell‐state regulation. To explore potential structural compatibility between disulfiram and VDAC1, we performed molecular docking as a hypothesis‐generating analysis (Figure S1B,C). The top‐ranked docking pose suggested plausible occupancy within a surface cavity of VDAC1, with predicted contact residues including Arg8, Tyr10, Asn127, Gly129, Thr119, Lys99, Asp131, and Ala144. These docking observations are intended to support structural hypotheses and do not constitute experimental evidence of direct intracellular binding. To provide an interpretable overview of the potential relationships between the nominated compound, the candidate node, and downstream biological programs, we summarized the connections between disulfiram, VDAC1, and enriched pathways using a Sankey diagram (Figure S1D). This visualization highlighted pathway modules including cholesterol metabolism, cellular senescence, necroptosis, and cGMP‐PKG signaling, suggesting potential links between mitochondrial regulation, metabolic processes, and regulated cell‐death programs. Consistent with a hub‐like role, coexpression analysis in the TCGA‐PAAD cohort showed that VDAC1 expression was positively correlated with CDK4, CDK6, and BCL2L1, while showing a modest negative correlation with BCL2 (Figure S1E–H). Together, these findings support VDAC1 as a potential central node within the nominated stress‐associated molecular network in PAAD.

### 3.3. A Disulfiram‐Nominated Gene Program Yields a Clinically Relevant Risk Signature

To evaluate whether the nominated gene set was associated with clinical outcomes, we constructed a LASSO‐Cox model based on genes derived from the nominated program (Figure S2A,B). The resulting risk score stratified patients into distinct prognostic groups across multiple survival endpoints. A random‐effects meta‐analysis integrating OS, DSS, DFI, and PFI showed a pooled hazard ratio of 7.43 without significant heterogeneity (Figure S2C), indicating a consistent association between higher risk scores and poorer clinical outcomes. Kaplan–Meier survival analyses further demonstrated significantly worse survival among high‐risk patients across all four survival endpoints (Figure S2D–G). Together, these findings indicate that the gene set derived from the nomination framework is associated with clinical prognosis in PAAD and supports further investigation of key nodes within this network.

### 3.4. VDAC1 Is Upregulated in PAAD and Associates With Diagnostic Separation and Adverse Outcomes

**Figure 2:**
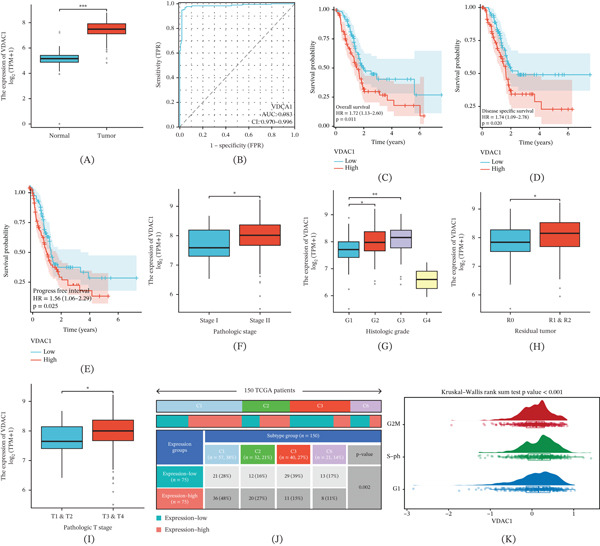
Expression patterns, diagnostic separation, and clinical associations of VDAC1 in pancreatic adenocarcinoma. (A) VDAC1 expression in PAAD tumors compared with normal pancreatic tissues. (B) Receiver operating characteristic (ROC) curve evaluating the diagnostic performance of VDAC1 in distinguishing tumor from normal samples (AUC = 0.983, 95% CI: 0.970–0.996). (C–E) Kaplan–Meier survival analyses comparing high‐ and low‐VDAC1 expression groups for (C) overall survival, (D) disease‐specific survival, (E) and progression‐free interval. (F–I) Associations between VDAC1 expression and clinicopathological characteristics, including (F) pathologic stage, (G) histologic grade, (H) residual tumor status, and (I) pathologic T stage in the TCGA‐PAAD cohort. (J) Distribution of TCGA immune subtypes between VDAC1 high‐ and low‐expression groups. (K) VDAC1 expression across inferred cell‐cycle phases (G1, S phase, and G2M); statistical significance assessed using the Kruskal–Wallis rank‐sum test.

VDAC1 was significantly upregulated in PAAD tumors compared with normal pancreatic tissues (Figure 2A). ROC analysis showed that VDAC1 had strong diagnostic performance for distinguishing PAAD tumors from normal pancreatic tissues, with an AUC of 0.983 (95% CI: 0.970–0.996) (Figure 2B). Clinically, elevated VDAC1 expression was associated with poorer OS, DSS, and PFI (Figure 2C–E). Specifically, high VDAC1 expression was associated with worse OS, DSS, and PFI. VDAC1 expression also showed significant associations with multiple clinicopathological characteristics, including pathologic stage, histologic grade, residual tumor status, and pathologic T stage (Figure 2F–I), indicating that elevated VDAC1 expression was associated with more advanced clinicopathological features. When stratified by TCGA immune subtypes, VDAC1 expression groups showed significantly different subtype distributions (Figure 2J), indicating an association between VDAC1 expression and immune subtype heterogeneity in PAAD. In addition, VDAC1 expression differed significantly across inferred cell‐cycle phases (Figure 2K), supporting an association with cell‐cycle status.

### 3.5. High VDAC1 Expression Marks Proliferative, Genome‐Maintenance, and Selective Metabolic Programs

To characterize pathway‐level programs associated with VDAC1, we first summarized pathway enrichment patterns across multiple independent PAAD cohorts using curated KEGG and Hallmark gene sets. A broadly consistent enrichment landscape was observed across datasets, indicating that VDAC1‐associated transcriptional programs were reproducible across cohorts (Figure S3A). Among the recurrently enriched signals, cell‐cycle‐related and stress‐associated programs were prominent in VDAC1‐high contexts. To further evaluate pathway‐level consistency across annotation systems, we performed integrated enrichment analysis using Reactome, WikiPathways, and GO collections. Pathways related to cell cycle, DNA replication, mitotic regulation, and checkpoint control were consistently enriched in the VDAC1‐high group, including Reactome Cell Cycle, Reactome M Phase, and DNA replication‐related terms (Figure S3B), supporting an association between elevated VDAC1 expression and genome‐maintenance programs. We next examined metabolic pathway activity by GSVA in the TCGA‐PAAD cohort. Distinct metabolic differences were observed between VDAC1‐high and VDAC1‐low tumors (Figure S3C). Pathways such as fructose and mannose metabolism, pentose and glucuronate interconversions, pentose phosphate pathway, pyruvate metabolism, and drug metabolism‐cytochrome P450 tended to be higher in the VDAC1‐high group, whereas pathways including lysine degradation and several glycosphingolipid‐related programs were relatively reduced, indicating selective rather than uniform metabolic activation. Finally, correlation analysis of curated oncogenic state signatures in an independent GEO cohort showed that VDAC1 expression was positively associated with cell cycle, DNA damage, DNA repair, hypoxia, and apoptosis scores, while negatively associated with angiogenesis, differentiation, inflammation, proliferation, and stemness‐related states (Figure S3D). These findings further support that VDAC1 is linked to reproducible pathway‐level alterations across independent datasets.

### 3.6. VDAC1‐High Tumors Exhibit a Coordinated Immune‐Suppressed Microenvironment

We next examined whether VDAC1 expression was associated with immune microenvironmental features in PAAD. Immune‐response modeling using the EaSIeR framework showed that several antitumor immune‐related features were reduced in VDAC1‐high tumors. Specifically, the MeTIL score was significantly lower in the VDAC1‐high group (Figure 3A). The chemokine‐related signature was also decreased in VDAC1‐high tumors (Figure 3B). CYT was similarly reduced in the VDAC1‐high group (Figure 3C). In contrast, the IFN‐*γ* signature did not differ significantly between groups (Figure 3D). Both the T cell‐inflamed signature and the TLS score were significantly lower in the VDAC1‐high group (Figure 3E,F). Together, these findings indicate that elevated VDAC1 expression is associated with reduced immune recruitment, diminished cytotoxic activity, and weaker lymphoid organization in PAAD. To further interpret these associations within the cancer‐immunity cycle, TIP‐based analyses showed that VDAC1 expression was mainly negatively correlated with multiple steps related to immune‐cell recruitment, tumor infiltration, recognition of cancer cells, and tumor‐cell killing (Figure 3G). Multialgorithm deconvolution analyses further indicated that VDAC1 expression tended to be negatively associated with several cytotoxic and antigen‐presentation–related immune populations, including CD8^+^ T‐cell, NK‐cell, and dendritic‐cell–related signatures across datasets and methods (Figure 3H,I). Group‐wise comparison further supported distinct immune landscapes between VDAC1‐high and VDAC1‐low tumors, with generally lower scores for multiple immune and microenvironment‐related components in the VDAC1‐high group (Figure 3J). Collectively, these results support VDAC1 as a transcriptomic feature associated with an immune‐suppressed PDAC microenvironment.

Figure 3Immune‐response modeling and immune infiltration patterns associated with VDAC1 expression in PAAD. (A) Comparison of MeTIL scores between VDAC1 high‐ and low‐expression groups. (B–F) Differences in EaSIeR‐derived immune response features, including (B) chemokine‐associated signature, (C) cytolytic activity (CYT), (D) interferon‐*γ* (IFN‐*γ*) signature, (E) T cell–inflamed signature, and (F) tertiary lymphoid structure (TLS) score. (G) Correlations between VDAC1 expression and cancer immunity cycle activities inferred by the TIP framework; red and blue squares denote positive and negative correlations, respectively, and symbol size reflects correlation strength. (H) Heatmap summarizing Spearman correlations between VDAC1 expression and immune cell infiltration estimates across multiple datasets and deconvolution algorithms; crosses indicate nonsignificant associations. (I) Summary dot plot showing algorithm‐wise Spearman correlation coefficients between VDAC1 expression and immune cell abundance. (J) Heatmap comparing immune infiltration patterns between VDAC1 high‐ and low‐expression groups.

**Figure:**
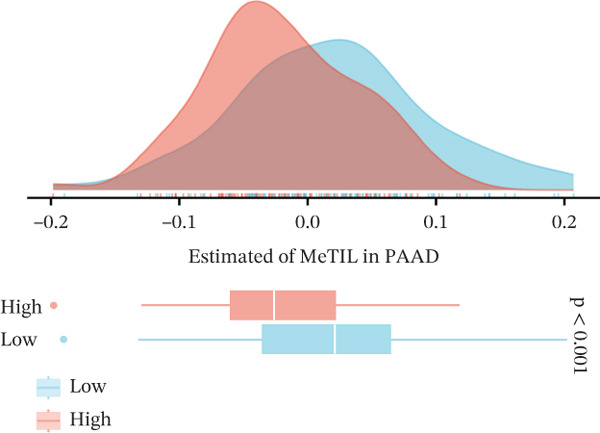
(A)

**Figure:**
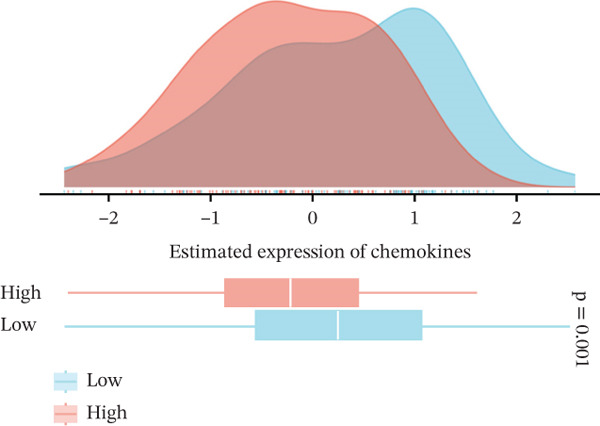
(B)

**Figure:**
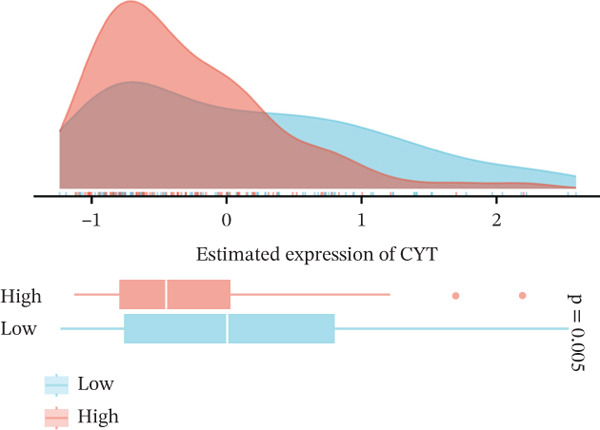
(C)

**Figure:**
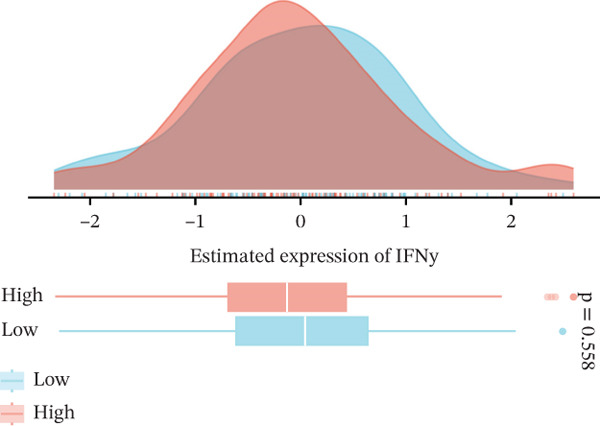
(D)

**Figure:**
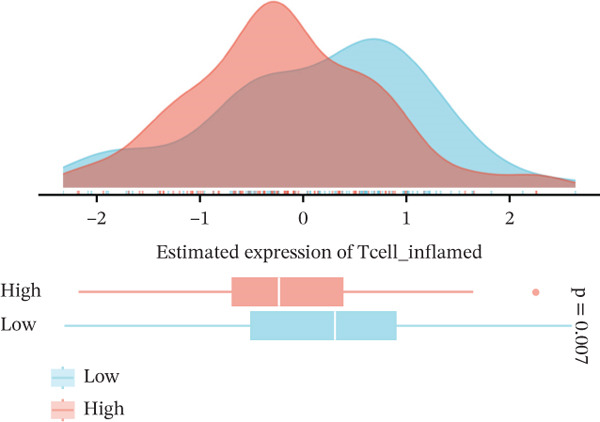
(E)

**Figure:**
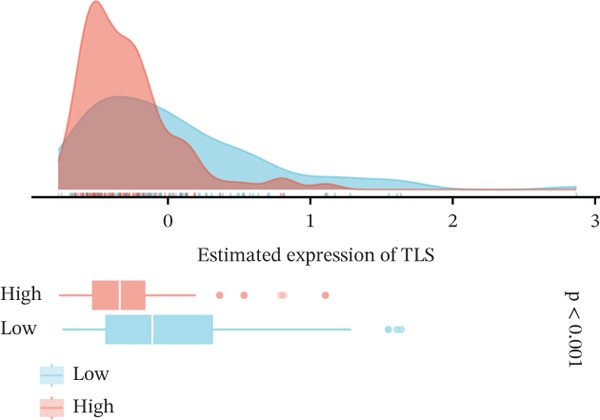
(F)

**Figure:**
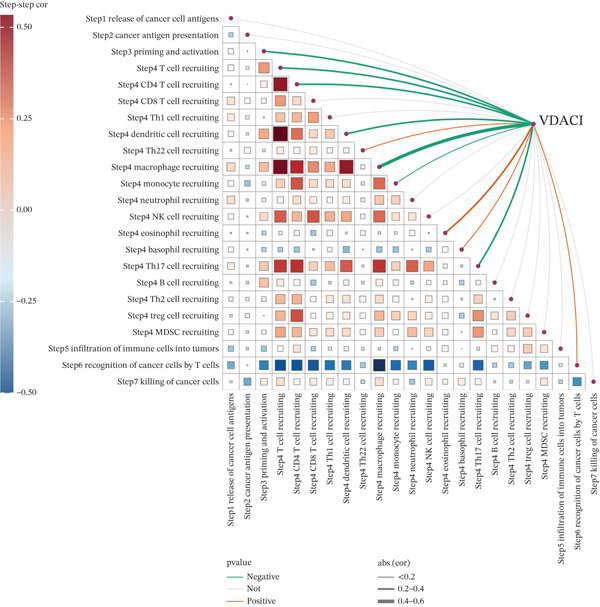
(G)

**Figure:**
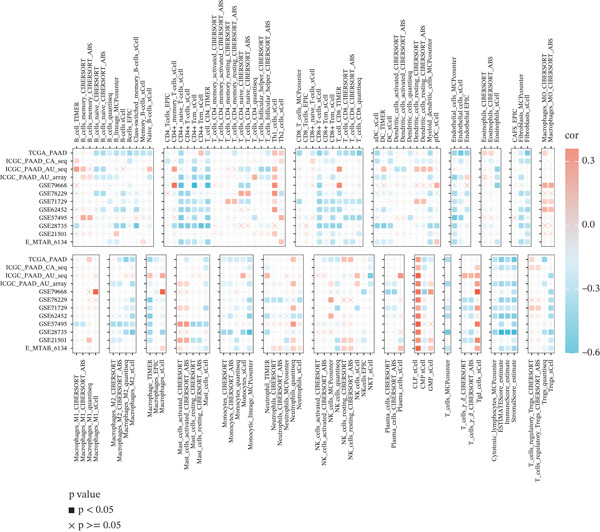
(H)

**Figure:**
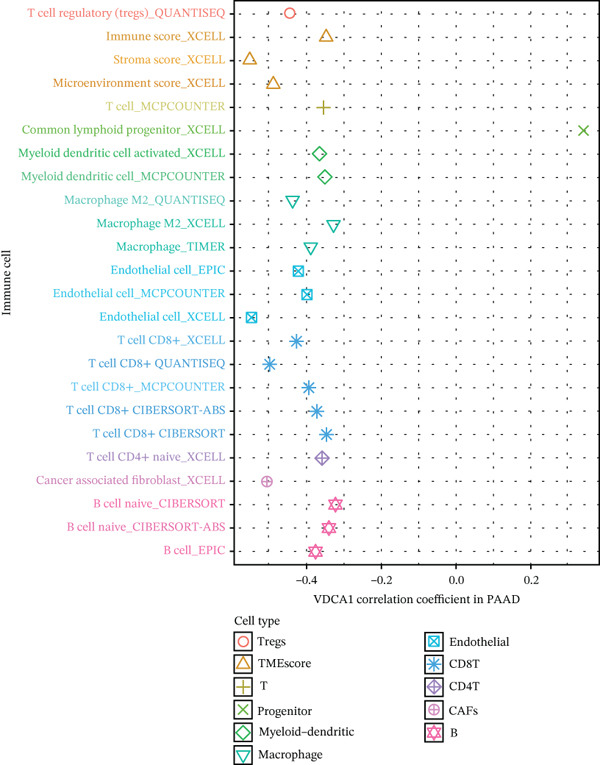
(I)

**Figure:**
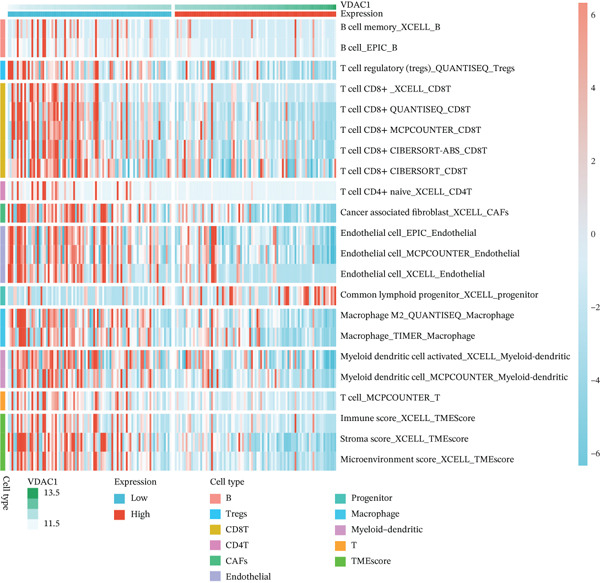
(J)

### 3.7. VDAC1 Expression Defines Selective Patterns of Resistance and Vulnerability in Pharmacogenomic Datasets

To connect VDAC1‐associated tumor states to therapy response patterns, we correlated VDAC1 expression with drug response profiles in CTRP and PRISM datasets. In CTRP, VDAC1 expression showed significant associations with responses to multiple compounds (Figure S4A). Higher VDAC1 expression was positively correlated with AUC values for several kinase inhibitors, including EGFR‐targeted agents such as canertinib, afatinib, and lapatinib, indicating reduced responsiveness in VDAC1‐high cell lines. Conversely, negative correlations were observed for selected compounds, including lovastatin and GDC‐0879, suggesting context‐specific vulnerabilities associated with VDAC1‐high states. In the independent PRISM dataset, broadly consistent trends were observed (Figure S4B). VDAC1 expression again exhibited positive correlations with AUC for multiple tyrosine kinase inhibitors (e.g., ibrutinib, erlotinib, gefitinib, neratinib, and osimertinib), supporting an association between VDAC1‐high states and reduced sensitivity to several targeted agents. In contrast, negative correlations for agents such as AMG‐232, abemaciclib, and nutlin‐3 indicated potential vulnerabilities in VDAC1‐high contexts. Together, these analyses suggest that VDAC1 expression tracks a drug‐response state characterized by selective resistance to subsets of kinase‐targeted therapies alongside distinct pharmacologic sensitivities.

### 3.8. Genomic Alteration and Copy Number Variation Characteristics of VDAC1 in PAAD

**Figure 4:**
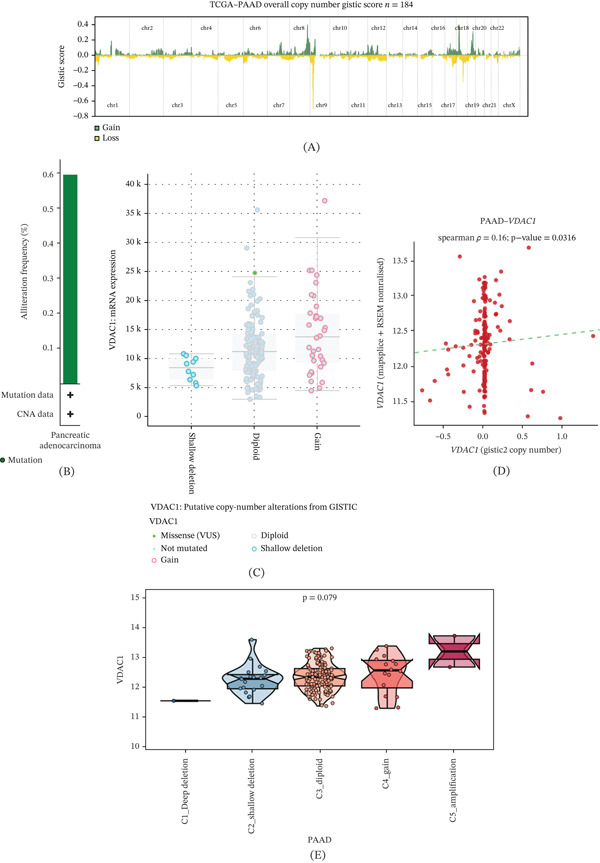
Genomic alteration and copy number variation characteristics of VDAC1 in pancreatic adenocarcinoma. (A) Overall copy number alteration landscape of the TCGA‐PAAD cohort based on GISTIC scores. Green indicates copy number gain and yellow indicates copy number loss. (B) Genomic alteration frequency of VDAC1 in PAAD, showing a low overall alteration rate of 0.6%. (C) Association between VDAC1 copy number status and mRNA expression in PAAD samples. (D) Correlation between VDAC1 GISTIC2 copy number values and normalized mRNA expression levels in PAAD. (E) Comparison of VDAC1 expression among different copy number subgroups, including deep deletion, shallow deletion, diploid, gain, and amplification.

To characterize the genomic features associated with VDAC1 in PAAD, we first examined the overall copy number alteration landscape of the TCGA‐PAAD cohort. As shown in Figure 4A, widespread chromosomal gains and losses were observed across the PAAD genome, indicating that copy number alteration is a common genomic event in this malignancy. We next evaluated the alteration frequency of VDAC1 in PAAD. VDAC1 showed an extremely low genomic alteration frequency, with mutation events detected in only 0.6% of cases, suggesting that recurrent somatic mutation is unlikely to be a major mechanism underlying VDAC1 dysregulation in pancreatic cancer (Figure 4B). Given the low mutation rate, we further explored the relationship between copy number status and VDAC1 expression. Samples with copy number gain tended to exhibit higher VDAC1 expression than diploid and shallow deletion groups in the cBioPortal‐based CNV‐expression comparison (Figure 4C). Consistently, correlation analysis demonstrated a weak but significant positive association between VDAC1 copy number and mRNA expression in PAAD (Figure 4D). We also compared VDAC1 expression across five copy number subgroups, including deep deletion, shallow deletion, diploid, gain, and amplification. Although the overall difference did not reach statistical significance, VDAC1 expression showed an increasing trend in the gain and amplification groups relative to deletion and diploid groups (Figure 4E). Collectively, these findings suggest that VDAC1 dysregulation in PAAD is characterized by a very low mutation frequency and may be partially associated with copy number alteration.

### 3.9. Tumor‐Intrinsic VDAC1 Supports Proliferative Capacity of Pancreatic Cancer Cells In Vitro

**Figure 5:**
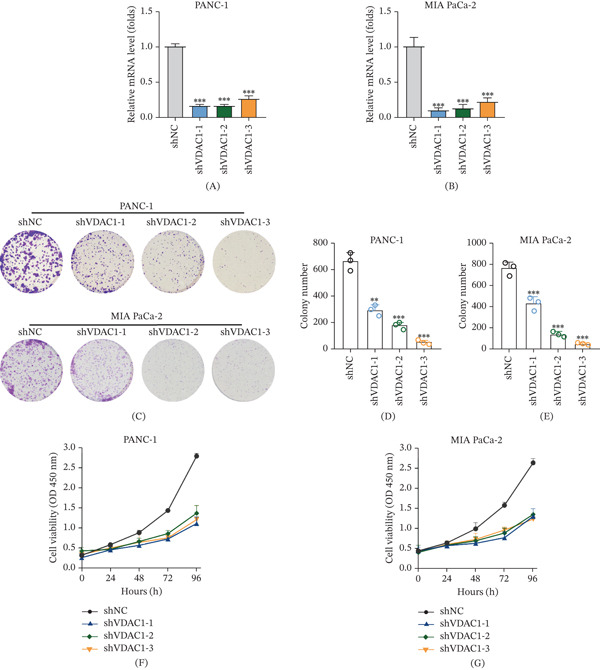
VDAC1 knockdown suppresses pancreatic cancer cell proliferation in vitro. (A–B) Relative VDAC1 mRNA levels in (A) PANC‐1 and (B) MIA PaCa‐2 cells after stable transduction with three independent shVDAC1 constructs (shVDAC1‐1/2/3), measured by quantitative PCR. (C) Representative colony formation images of PANC‐1 and MIA PaCa‐2 cells following VDAC1 knockdown. (D–E) Quantification of colony numbers in (D) PANC‐1 and (E) MIA PaCa‐2 cells. (F–G) CCK‐8 assays showing cell viability of (F) PANC‐1 and (G) MIA PaCa‐2 cells at the indicated time points. Data are presented as mean ± SD from three independent experiments.

Stable VDAC1 knockdown was established in PANC‐1 and MIA PaCa‐2 and confirmed by quantitative PCR, with all three shRNA constructs producing substantial reductions in VDAC1 mRNA levels compared with control cells (Figure 5A,B). Functionally, VDAC1 depletion impaired clonogenic growth in both PANC‐1 and MIA PaCa‐2 cells (Figure 5C–E). Representative colony‐formation images showed markedly reduced colony formation following VDAC1 knockdown, and quantitative analyses confirmed significant decreases in colony numbers across multiple shVDAC1 constructs. Consistent with these findings, short‐term proliferation assays demonstrated reduced growth rates over time. CCK‐8 assays showed lower OD450 values in VDAC1‐silenced cells compared with shNC controls across multiple time points in both PANC‐1 and MIA PaCa‐2 models (Figure 5F,G). Together, these results indicate that VDAC1 contributes to the proliferative capacity of pancreatic cancer cells in vitro.

### 3.10. VDAC1 Knockdown Compromises Mitochondrial Bioenergetics and Reduces mtROS Signals in Pancreatic Cancer Cells

**Figure 6:**
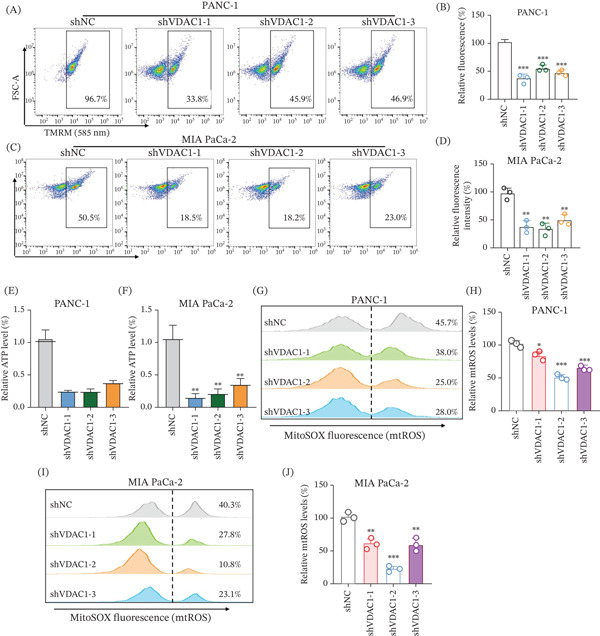
VDAC1 knockdown impairs mitochondrial bioenergetics and reduces mitochondrial ROS signals in pancreatic cancer cells. (A) Representative flow cytometry plots of TMRM staining showing reduced mitochondrial membrane potential (*ΔΨ*m) in PANC‐1 cells after VDAC1 knockdown (shVDAC1‐1/2/3) compared with shNC controls. (B) Quantification of relative TMRM fluorescence intensity in PANC‐1 cells corresponding to Panel A. (C) Representative TMRM flow cytometry plots in MIA PaCa‐2 cells following VDAC1 knockdown. (D) Quantification of relative TMRM fluorescence intensity in MIA PaCa‐2 cells corresponding to Panel C. (E–F) Intracellular ATP levels in (E) PANC‐1 and (F) MIA PaCa‐2 cells measured using the Cell‐ATP Viability Detection Kit, showing reduced ATP levels following VDAC1 silencing. (G, I) Representative MitoSOX Red flow cytometry distributions in (G) PANC‐1 and (I) MIA PaCa‐2 cells indicating reduced mitochondrial ROS (mtROS) signals after VDAC1 knockdown, with dashed lines denoting the gating threshold and percentages indicating MitoSOX‐high fractions. (H, J) Quantification of relative mtROS levels in (H) PANC‐1 and (J) MIA PaCa‐2 cells. Data are presented as mean ± SD from three independent experiments; statistical significance was determined compared with shNC controls.

To determine whether VDAC1 is involved in maintaining mitochondrial energetic homeostasis in pancreatic cancer cells, we quantified mitochondrial membrane potential, ATP production, and mtROS‐associated signals following stable VDAC1 silencing. Flow cytometric analysis using TMRM staining showed a marked decrease in mitochondrial membrane potential (*ΔΨ*m) in both PANC‐1 and MIA PaCa‐2 cells after VDAC1 knockdown, as reflected by reduced TMRM‐high fractions and lower relative fluorescence intensity across the three shVDAC1 constructs compared with shNC controls (Figure 6A–D). Intracellular ATP levels were also reduced after VDAC1 depletion in both cell lines, with statistically significant decreases observed in MIA PaCa‐2 cells (Figure 6E,F). We next assessed mitochondrial oxidative status using MitoSOX Red and found reduced mtROS‐associated fluorescence signals following VDAC1 silencing, accompanied by decreased MitoSOX‐high fractions and corresponding quantitative reductions in both PANC‐1 and MIA PaCa‐2 cells (Figure 6G–J). Together, these results indicate that VDAC1 contributes to maintaining mitochondrial bioenergetic function and redox‐related signaling in pancreatic cancer cells, and that its depletion is accompanied by coordinated reductions in *ΔΨ*m, ATP levels, and mtROS signals.

### 3.11. Tumor‐Intrinsic VDAC1 Perturbation Reshapes Immune‐Facing Programs and Reduces Immunosuppressive Cytokine Output

To examine whether tumor‐intrinsic mitochondrial gatekeeping by VDAC1 intersects with immune evasion‐relevant programs, we quantified immune checkpoint‐related transcripts and immunosuppressive cytokine levels following VDAC1 knockdown in pancreatic cancer cells. As shown in Figure S5A,B, silencing VDAC1 significantly reduced PD‐L1 expression in both PANC‐1 and MIA PaCa‐2 cells compared with shNC controls, indicating that mitochondrial perturbation can remodel checkpoint‐associated programs. In parallel, VDAC1 knockdown also reduced HLA‐A expression in both cell lines (Figure S5C,D), suggesting that mitochondrial state is coupled to antigen presentation‐related machinery. Because MHC‐I‐associated regulation is highly context dependent and expression alone does not uniquely define functional antigen presentation capacity, we interpret this change as part of a broader immune‐related program shift rather than assigning a single‐direction functional consequence. We next assessed whether VDAC1 affects tumor‐derived soluble factors that contribute to immune restriction. ELISA measurements showed that VDAC1 depletion significantly reduced levels of the immunosuppressive cytokines TGF‐*β* and IL‐10 in both PANC‐1 and MIA PaCa‐2 cells (Figure S5E–H). Together, these results indicate that tumor‐intrinsic VDAC1 supports an immune‐relevant state characterized by coordinated regulation of checkpoint‐ and antigen presentation‐associated programs and the output of suppressive cytokines, suggesting a potential link between mitochondrial metabolic control and immune microenvironmental remodeling in pancreatic cancer.

## 4. Discussion

This study nominates VDAC1 as a mitochondrial–immune coupling node in PAAD and places it within an integrated framework of metabolic adaptation, TME organization, and therapy‐refractory phenotypes. By combining bulk transcriptomics, genomic alteration characterization, immune‐response modeling, and pharmacogenomic associations, we identify a reproducible VDAC1‐high state characterized by enhanced proliferative and mitochondrial programs together with attenuated cytotoxic immune features. Genomic analyses further indicated that VDAC1 harbors a low mutation frequency and that its transcriptional dysregulation may be partly associated with copy number alterations in PAAD. Complementing these computational observations, tumor‐intrinsic VDAC1 perturbation produced convergent phenotypes—including impaired mitochondrial function, suppressed tumor growth, and remodeling of immune‐related readouts—supporting the view that VDAC1 represents a biologically interpretable biomarker linked to resistance‐associated tumor states in PAAD.

A key mechanistic implication of our findings is that VDAC1‐dependent mitochondrial state aligns tightly with malignant fitness under stress. VDAC1 is widely recognized as a dominant outer mitochondrial membrane conduit for metabolite flux, enabling coupling between cytosolic inputs and mitochondrial bioenergetics through interactions with hexokinase and other metabolic regulators [57, 58]. In our models, VDAC1 silencing consistently reduced clonogenicity and proliferative capacity, accompanied by diminished mitochondrial membrane potential, ATP output, and altered mitochondrial redox‐associated readouts. These phenotypes are coherent with a broader literature in which mitochondrial “fitness” supports sustained growth under nutrient limitation and therapeutic stress—conditions particularly salient in PAAD, where hypoperfusion, elevated interstitial pressure, and stromal sequestration generate chronic metabolic constraints [59–61]. The concordance between mitochondrial impairment and growth suppression across multiple cell systems argues that VDAC1 lies upstream of a bioenergetic dependency that PAAD cells exploit to maintain tumor‐initiating potential.

Beyond tumor‐intrinsic fitness, our results suggest that mitochondrial gatekeeping intersects with immune‐facing tumor programs, but in a manner that is best interpreted as immune remodeling rather than unidirectional “immune activation.” We observed reduced PD‐L1 expression following VDAC1 knockdown, indicating that mitochondrial state can influence checkpoint‐associated surface programs [62, 63]. Prior work across solid tumors has linked metabolic stress and redox signaling to PD‐L1 regulation through stress‐responsive transcriptional and posttranscriptional pathways; within this conceptual frame, the mitochondrial disruptions after VDAC1 perturbation provide a plausible upstream route to altered checkpoint programs. We also detected changes in HLA‐A expression [64], implicating the antigen‐presentation axis as another interface sensitive to mitochondrial state. Importantly, the observed directionality does not support a simplistic interpretation that VDAC1 depletion universally “improves” antigen presentation; rather, it indicates that VDAC1 perturbation reconfigures immune visibility‐related machinery, potentially through stress adaptation, proteostasis, or transcriptional state shifts. Dissecting the causal intermediates that connect mitochondrial regulation to PD‐L1 and HLA‐A dynamics will be essential to determine when these changes facilitate immune recognition versus immune escape.

Consistent with immune remodeling, tumor‐intrinsic VDAC1 depletion reduced secretion of TGF‐*β* and IL‐10, two canonical immunosuppressive mediators that can constrain cytotoxic T‐cell recruitment and effector function in PAAD [65, 66]. These cytokines are typically viewed as outputs of tumor–stroma coevolution that help maintain an immune‐restricted niche. Our observation that reducing VDAC1 in tumor cells alone was sufficient to lower these factors supports a model in which malignant metabolic wiring can actively contribute to the immunoregulatory milieu, rather than being merely shaped by stromal cues. Notably, in this revised study our experimental validation is restricted to tumor cell–intrinsic assays; therefore, any immune‐cell functional consequences should be interpreted as hypothesis generating and anchored primarily by bulk immune deconvolution/signature analyses. Future work incorporating immune‐context models will be required to establish causal links between VDAC1 modulation, immune infiltration, and effector competence [67].

The pharmacogenomic correlations provide a translational complement by linking VDAC1 expression to selective drug‐response patterns across large‐scale screens [68, 69]. Resistance in PAAD is multifactorial, reflecting tumor‐intrinsic stress tolerance, apoptotic thresholding, and DNA damage responses, compounded by delivery barriers imposed by desmoplasia and immune‐mediated selection pressures. VDAC1 is plausibly positioned at the convergence of these pressures: mitochondrial flux and redox balance influence susceptibility to regulated cell death, whereas mitochondrial state can shape tumor–stroma interactions and immune suppression. Although correlative associations cannot establish causality, their concordance with the robust phenotypes observed after VDAC1 perturbation supports the interpretation that VDAC1 may represent a vulnerability rather than a purely descriptive biomarker.

Several limitations define immediate next steps. First, immune deconvolution and signature inference from bulk RNA‐seq provide indirect estimates of microenvironmental composition and state [70, 71], and cannot fully resolve spatial organization or causal ordering. Second, our experimental work emphasizes phenotypic endpoints; mechanistic mapping of the signaling nodes connecting mitochondrial state to checkpoint/antigen‐presentation programs and cytokine output will be required to establish direct pathways. Third, as VDAC1 is a ubiquitous mitochondrial channel, systemic inhibition raises therapeutic‐index considerations [72], motivating strategies that optimize delivery, context dependence, and combination regimens. Finally, our functional validation focuses on tumor cell–intrinsic phenotypic endpoints; orthogonal immune‐interaction and spatially resolved approaches would strengthen mechanistic inference and clarify how tumor‐intrinsic mitochondrial rewiring translates into microenvironmental remodeling.

Finally, several limitations of this study should be acknowledged. First, although multicohort transcriptomic and genomic analyses support the association between VDAC1 and adverse molecular programs in PAAD, the retrospective nature of public datasets may introduce inherent selection biases. Second, although our functional assays demonstrate that VDAC1 contributes to mitochondrial homeostasis and proliferative fitness, the detailed molecular mechanisms linking VDAC1‐mediated metabolic regulation to immune remodeling require further investigation. Third, additional in vivo validation and larger clinical cohorts will be necessary to clarify the translational relevance of VDAC1‐associated molecular alterations. Despite these limitations, the integrative design combining genomic alteration profiling, transcriptomic analyses, and functional validation provides convergent evidence supporting VDAC1 as a biologically relevant mitochondrial regulator associated with tumor progression and immune context in pancreatic cancer.

## 5. Conclusion

In summary, integrative multiomics analyses combined with functional experiments identify VDAC1 as a mitochondrial regulator associated with metabolic and immune‐related features in PDAC. VDAC1 silencing impaired mitochondrial function, suppressed tumor cell proliferation, and reduced immunosuppressive cytokine expression in vitro. These findings support VDAC1 as a candidate molecular biomarker linked to mitochondrial metabolic regulation in PDAC.

## Author Contributions

Y.T., L.C., and B.Z. designed and conceptualized the study and supervised the project. W.S., Y.W., B.Z., and X.Q. performed the computational analyses and data visualization. W.S., Y.W., X.Q., S.Z., Z.L., S.Y., B.Z., and D.Z. conducted the in vitro experiments. D.W., H.Z., F.Q., M.Z., B.Z., and B.W. contributed to data curation, statistical analyses, and figure preparation. W.S., Y.W., X.Q., S.Z., Z.L., S.Y., D.Z., D.W., H.Z., F.Q., M.Z., B.Z., and B.W. drafted the manuscript. Y.T., L.C., and B.Z. critically revised the manuscript for important intellectual content and provided manuscript editing. W.S., Y.W., and X.Q., have contributed equally to this study.

## Funding

This study was supported by Anhui Province Translational Medicine Research Project (202427b10020020); Natural Science Key Project of Bengbu Medical University (2022byzd050); Anhui Provincial Department of Education Natural Science Research Youth Project (2025AHGXZK40703); Bengbu Medical University “Jie Bang Gua Shuai” Research Project (2025byjbgs064).

## Disclosure

The study conception and design, data acquisition, analysis, interpretation, and all scientific conclusions were developed by the authors, who take full responsibility for the integrity and accuracy of the work. All authors reviewed and approved the final version of the manuscript.

## Conflicts of Interest

The authors declare no conflicts of interest.

## Supporting Information

Additional supporting information can be found online in the Supporting Information section.

## Supporting information


**Supporting Information 1** Figure S1 Multilevel prioritization of VDAC1 as a candidate core node within the nominated network. (A) VDAC1‐centered local protein–protein interaction subnetwork showing VDAC1 and its first‐order interactors. (B) Surface representation of VDAC1 with the predicted docking pocket for disulfiram based on in silico analysis. (C) Representative docking pose showing a plausible disulfiram–VDAC1 configuration and annotated contact residues; docking is presented as hypothesis generating and does not demonstrate cellular binding. (D) Sankey diagram illustrating the connections between disulfiram, VDAC1, and enriched pathways derived from the candidate gene set. (E–H) Coexpression relationships between VDAC1 and (E) CDK6, (F) BCL2L1, (G) BCL2, and (H) CDK4 in the TCGA‐PAAD cohort.

**Supporting Information 2** Figure 2: Construction and prognostic evaluation of the nominated gene signature. (A) Tenfold cross‐validation plot showing partial likelihood deviance for selection of the optimal penalty parameter (*λ*) in the LASSO–Cox model. (B) LASSO coefficient profiles of genes included in the model across different values of log(*λ*). (C) Forest plot summarizing the random‐effects meta‐analysis of the derived risk score across survival endpoints (*I* ^2^ = 0*%*). (D–G) Kaplan–Meier survival curves comparing high‐ and low‐risk groups for (D) disease‐free interval (DFI), (E) disease‐specific survival (DSS), (F) overall survival (OS), and (G) progression‐free interval (PFI).

**Supporting Information 3** Figure 3: Pathway‐level transcriptional and metabolic programs associated with VDAC1 expression in PAAD. (A) Integrated bubble plot summarizing enrichment patterns of curated KEGG and Hallmark pathways across multiple independent PAAD cohorts. Bubble color indicates normalized enrichment score (NES) and bubble size. (B) Consensus enrichment results across Reactome, WikiPathways, and Gene Ontology collections, highlighting pathways associated with VDAC1 expression. (C) GSVA‐based comparison of metabolic pathway activity between VDAC1‐high and VDAC1‐low tumors in the TCGA‐PAAD cohort. Positive *t* values indicate pathways enriched in the VDAC1‐high group, whereas negative *t* values indicate pathways enriched in the VDAC1‐low group. (D) Correlation analysis between VDAC1 expression and curated oncogenic state scores in an independent GEO cohort.

**Supporting Information 4** Figure 4: Association between VDAC1 expression and drug sensitivity in CTRP and PRISM datasets. (A) Lollipop plot showing Spearman correlation coefficients (*ρ*) between VDAC1 expression and drug AUC values in CTRP. (B) Lollipop plot showing Spearman correlation coefficients (*ρ*) between VDAC1 expression and drug AUC values in PRISM. Each point represents a compound, with bar length indicating correlation magnitude. Positive correlations indicate higher AUC values (reduced sensitivity) associated with elevated VDAC1 expression, whereas negative correlations indicate lower AUC values (increased sensitivity). Compounds are ranked by correlation coefficient, with corresponding *p* values shown.

**Supporting Information 5** Figure 5: Tumor‐intrinsic VDAC1 perturbation remodels immune‐related programs and reduces immunosuppressive cytokine output in pancreatic cancer cells. (A–B) Relative PD‐L1 expression in (A) PANC‐1 and (B) MIA PaCa‐2 cells following VDAC1 knockdown with three independent shVDAC1 constructs versus shNC controls. (C–D) Reduced HLA‐A expression in (C) PANC‐1 and (D) MIA PaCa‐2 cells after VDAC1 silencing, indicating remodeling of antigen presentation–related programs. (E–H) ELISA quantification showing reduced levels of TGF‐*β* in (E) PANC‐1 and (F) MIA PaCa‐2 cells, and reduced IL‐10 levels in (G) PANC‐1 and (H) MIA PaCa‐2 cells following VDAC1 knockdown. Data are shown as mean ± SD; significance was determined compared with shNC controls.

**Supporting Information 6** Table S1: Primer sequences.

## Data Availability

All datasets used in this study are publicly available. TCGA data were obtained from the Genomic Data Commons portal, GEO datasets from the NCBI GEO repository, and pharmacogenomic data from CTRP and PRISM via the DepMap portal. Additional information is available from the corresponding authors upon reasonable request.
