# Supplementary figures and images for: Multiomics and Genomic Alteration Characterization Identifies VDAC1 as a Mitochondrial‐Associated Biomarker in Pancreatic Cancer
Source: Hum Mutat. 2026 May 6;2026:5510306. doi: 10.1155/humu/5510306 (PMC13150345; doi:10.1155/humu/5510306)

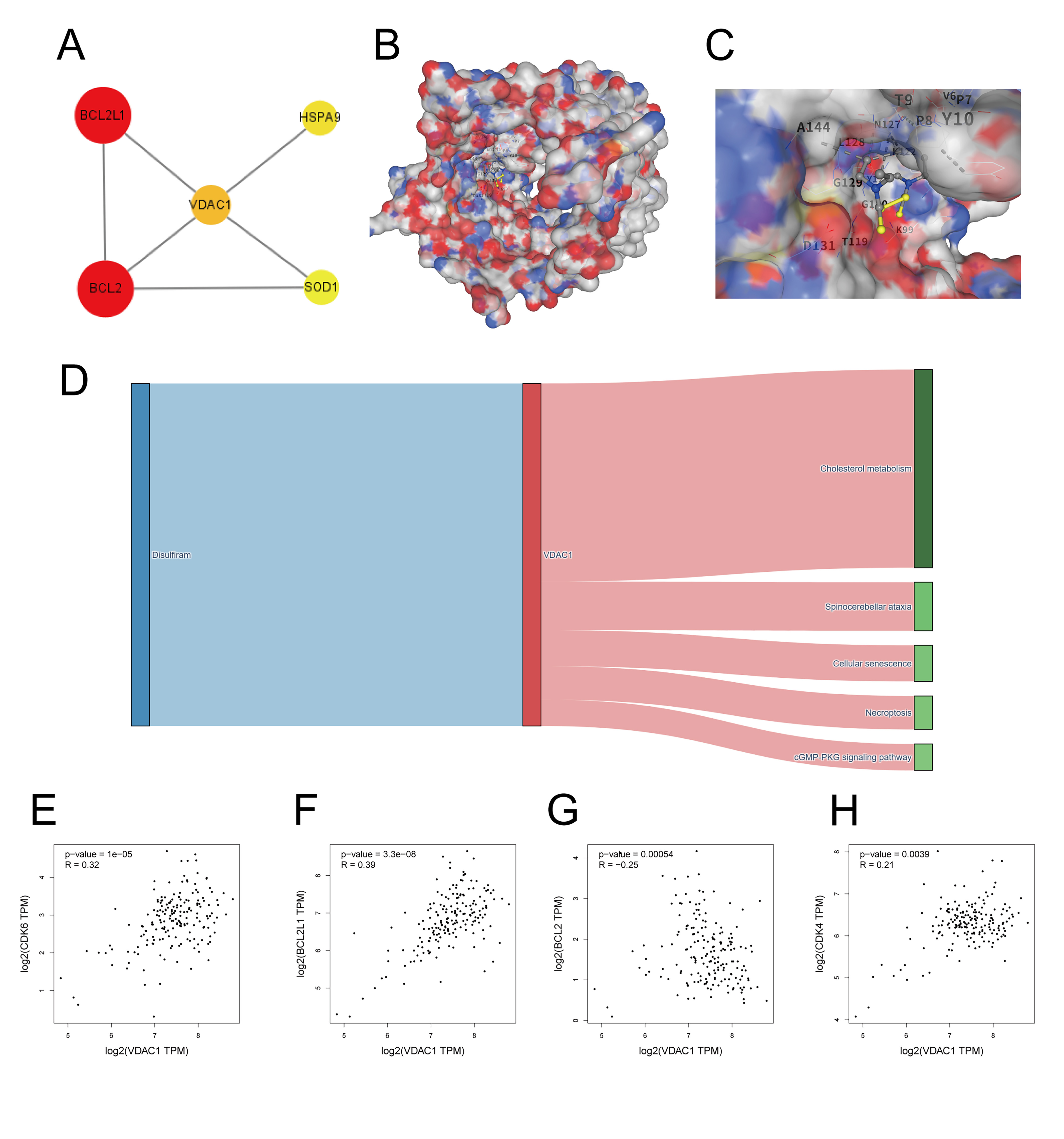

Supplement: Supplementary file 1 — Supporting Information 1 Figure S1 Multilevel prioritization of VDAC1 as a candidate core node within the nominated network. (A) VDAC1‐centered local protein–protein interaction subnetwork showing VDAC1 and its first‐order interactors. (B) Surface representation of VDAC1 with the predicted docking pocket for disulfiram based on in silico analysis. (C) Representative docking pose showing a plausible disulfiram–VDAC1 configuration and annotated contact residues; docking is presented as hypothesis generating and does not demonstrate cellular binding. (D) Sankey diagram illustrating the connections between disulfiram, VDAC1, and enriched pathways derived from the candidate gene set. (E–H) Coexpression relationships between VDAC1 and (E) CDK6, (F) BCL2L1, (G) BCL2, and (H) CDK4 in the TCGA‐PAAD cohort. [file HUMU-2026-5510306-s003.jpg]

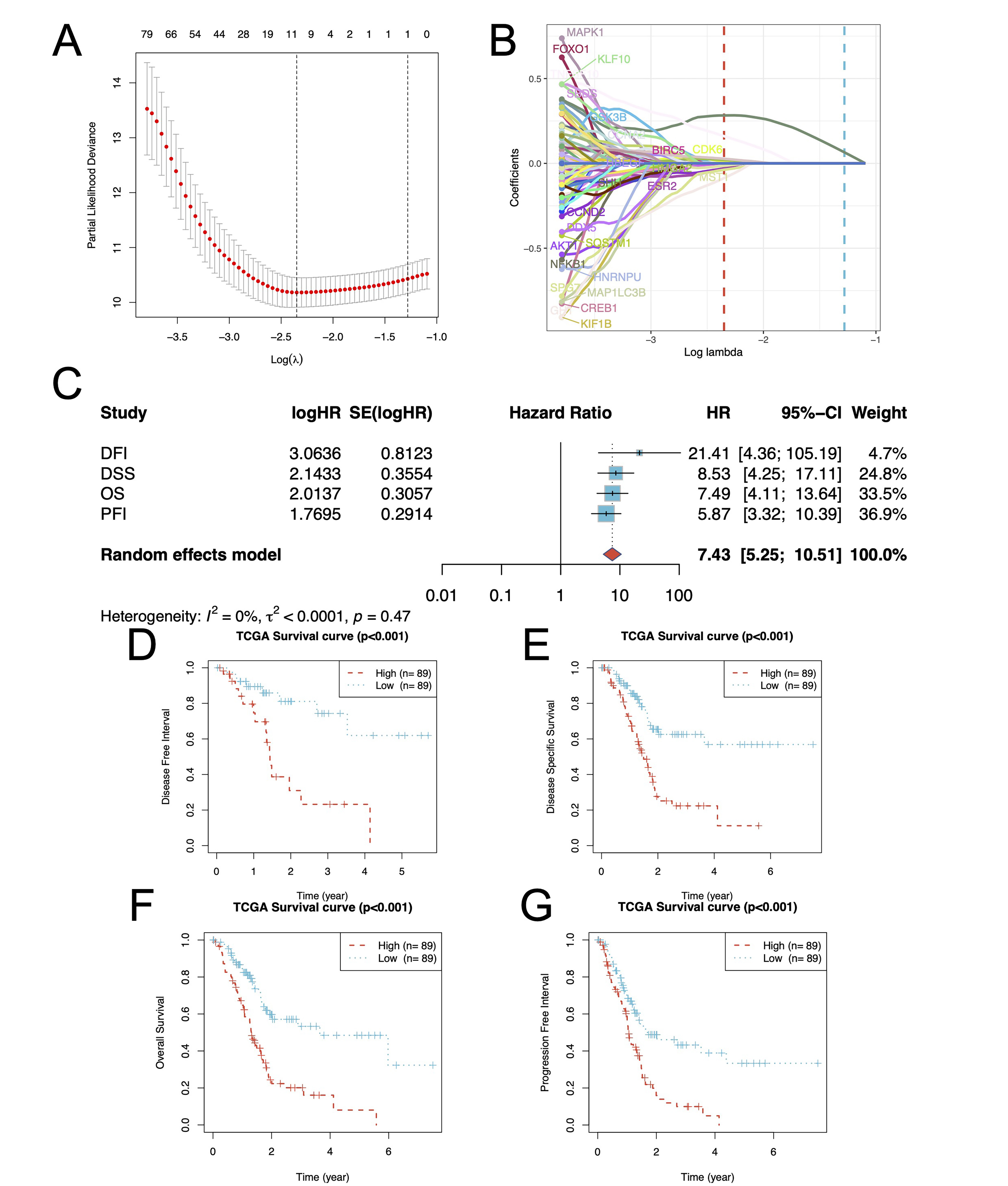

Supplement: Supplementary file 2 — Supporting Information 2 Figure 2: Construction and prognostic evaluation of the nominated gene signature. (A) Tenfold cross‐validation plot showing partial likelihood deviance for selection of the optimal penalty parameter (λ) in the LASSO–Cox model. (B) LASSO coefficient profiles of genes included in the model across different values of log(λ). (C) Forest plot summarizing the random‐effects meta‐analysis of the derived risk score across survival endpoints (I 2 = 0%). (D–G) Kaplan–Meier survival curves comparing high‐ and low‐risk groups for (D) disease‐free interval (DFI), (E) disease‐specific survival (DSS), (F) overall survival (OS), and (G) progression‐free interval (PFI). [file HUMU-2026-5510306-s001.jpg]

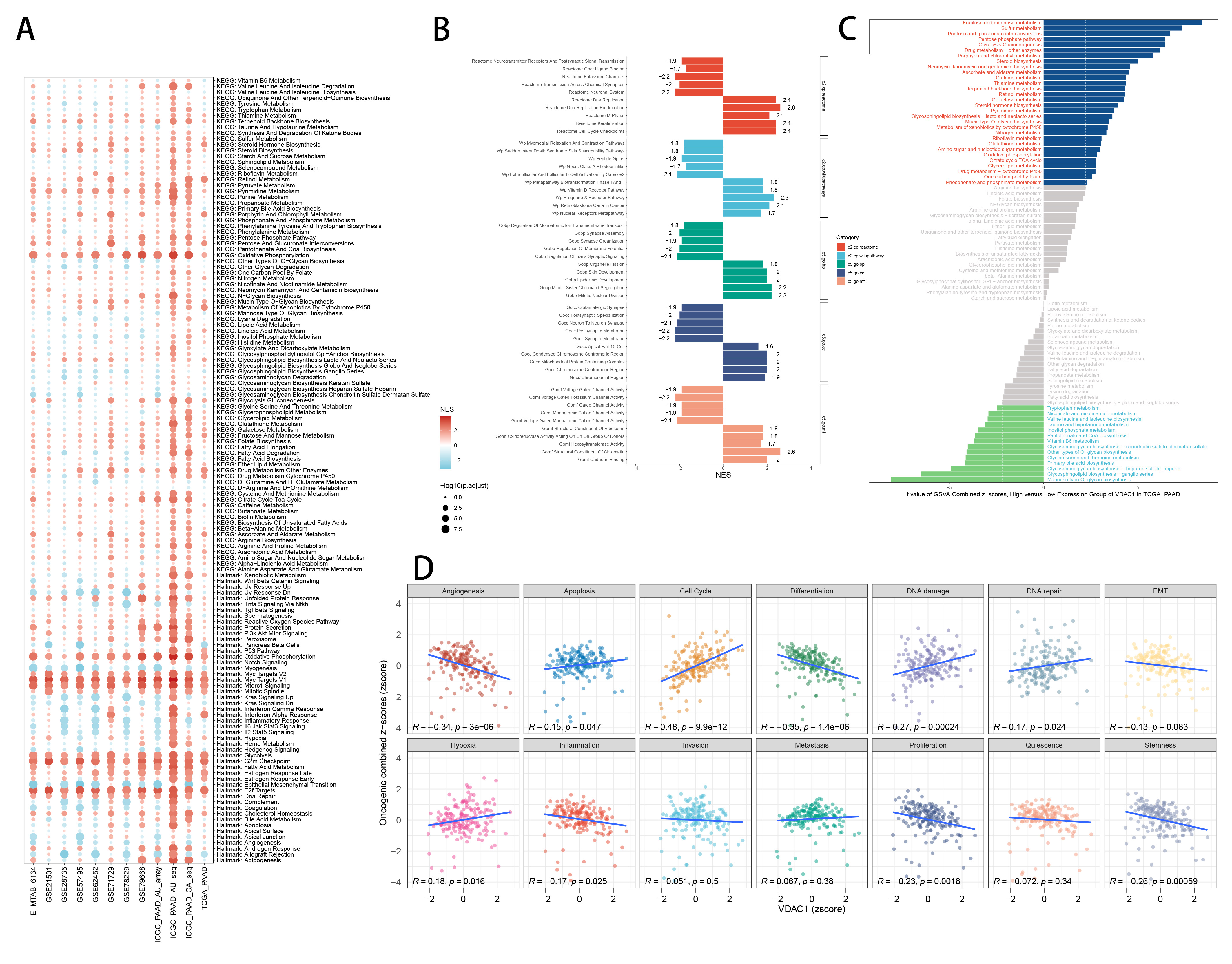

Supplement: Supplementary file 3 — Supporting Information 3 Figure 3: Pathway‐level transcriptional and metabolic programs associated with VDAC1 expression in PAAD. (A) Integrated bubble plot summarizing enrichment patterns of curated KEGG and Hallmark pathways across multiple independent PAAD cohorts. Bubble color indicates normalized enrichment score (NES) and bubble size. (B) Consensus enrichment results across Reactome, WikiPathways, and Gene Ontology collections, highlighting pathways associated with VDAC1 expression. (C) GSVA‐based comparison of metabolic pathway activity between VDAC1‐high and VDAC1‐low tumors in the TCGA‐PAAD cohort. Positive t values indicate pathways enriched in the VDAC1‐high group, whereas negative t values indicate pathways enriched in the VDAC1‐low group. (D) Correlation analysis between VDAC1 expression and curated oncogenic state scores in an independent GEO cohort. [file HUMU-2026-5510306-s002.jpg]

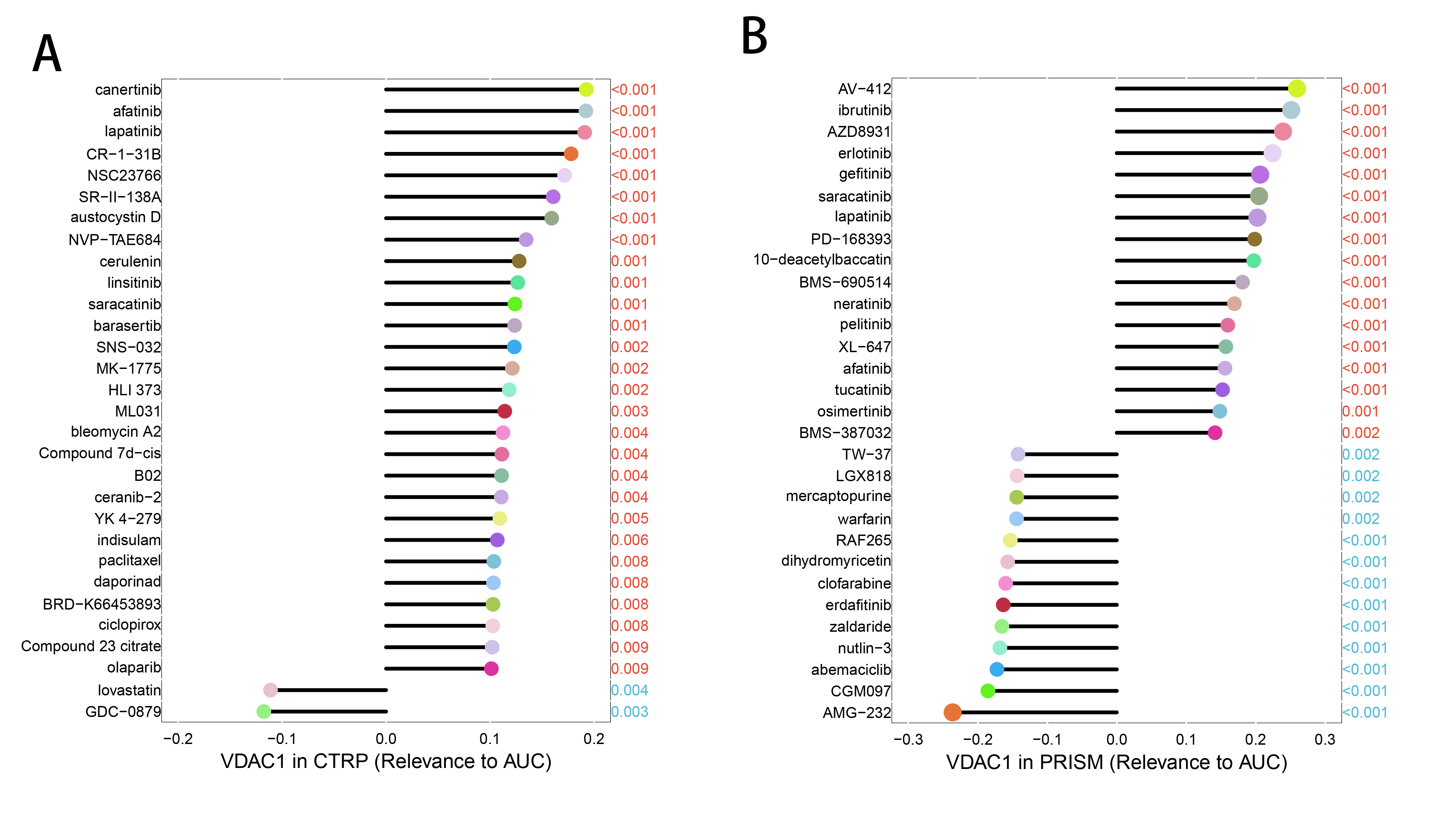

Supplement: Supplementary file 4 — Supporting Information 4 Figure 4: Association between VDAC1 expression and drug sensitivity in CTRP and PRISM datasets. (A) Lollipop plot showing Spearman correlation coefficients (ρ) between VDAC1 expression and drug AUC values in CTRP. (B) Lollipop plot showing Spearman correlation coefficients (ρ) between VDAC1 expression and drug AUC values in PRISM. Each point represents a compound, with bar length indicating correlation magnitude. Positive correlations indicate higher AUC values (reduced sensitivity) associated with elevated VDAC1 expression, whereas negative correlations indicate lower AUC values (increased sensitivity). Compounds are ranked by correlation coefficient, with corresponding p values shown. [file HUMU-2026-5510306-s006.jpg]

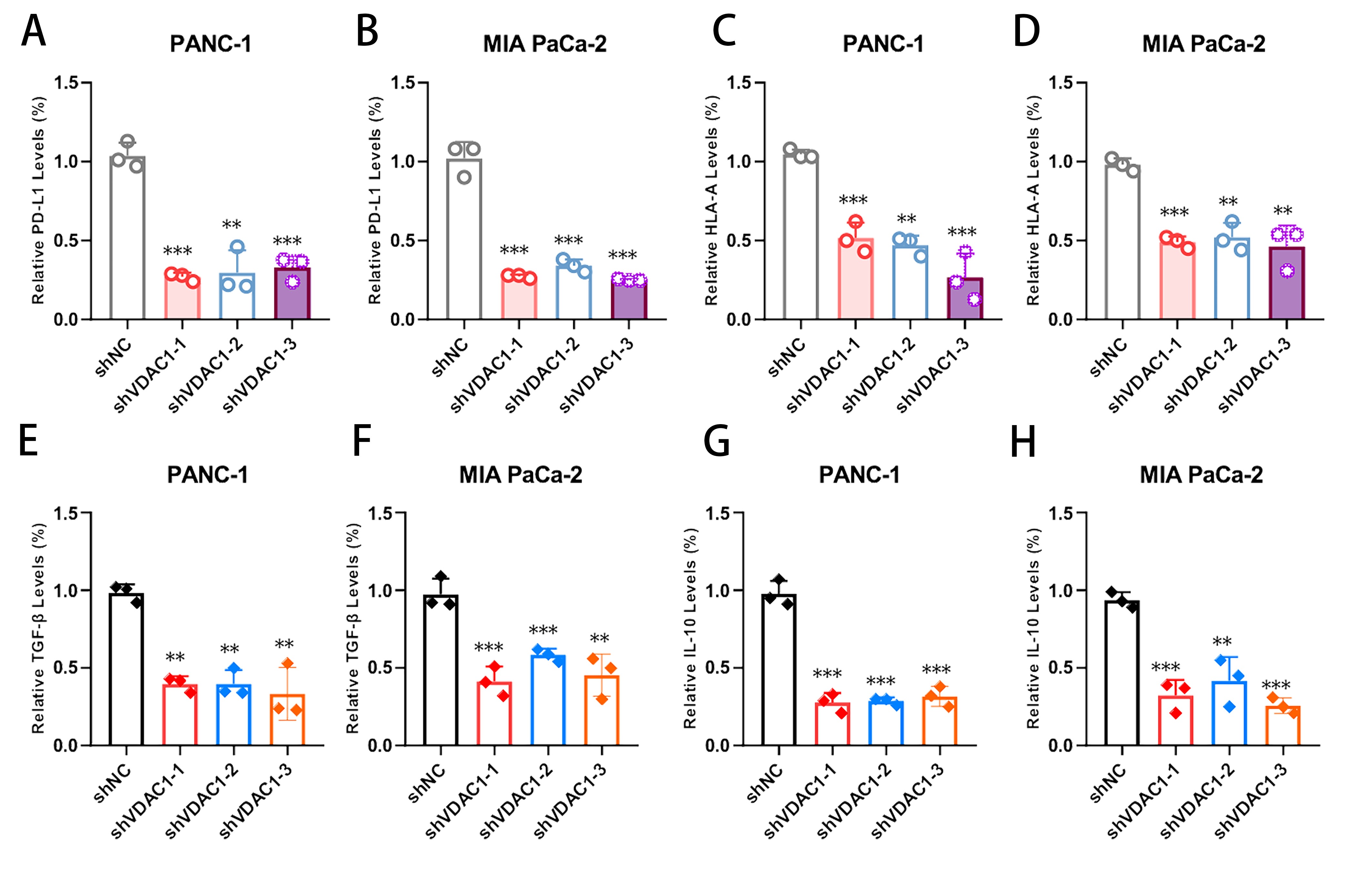

Supplement: Supplementary file 5 — Supporting Information 5 Figure 5: Tumor‐intrinsic VDAC1 perturbation remodels immune‐related programs and reduces immunosuppressive cytokine output in pancreatic cancer cells. (A–B) Relative PD‐L1 expression in (A) PANC‐1 and (B) MIA PaCa‐2 cells following VDAC1 knockdown with three independent shVDAC1 constructs versus shNC controls. (C–D) Reduced HLA‐A expression in (C) PANC‐1 and (D) MIA PaCa‐2 cells after VDAC1 silencing, indicating remodeling of antigen presentation–related programs. (E–H) ELISA quantification showing reduced levels of TGF‐β in (E) PANC‐1 and (F) MIA PaCa‐2 cells, and reduced IL‐10 levels in (G) PANC‐1 and (H) MIA PaCa‐2 cells following VDAC1 knockdown. Data are shown as mean ± SD; significance was determined compared with shNC controls. [file HUMU-2026-5510306-s004.jpg]
